# Proteomic profiling of ascidians as a tool for biomonitoring marine environments

**DOI:** 10.1371/journal.pone.0215005

**Published:** 2019-04-09

**Authors:** Zafrir Kuplik, Lion Novak, Noa Shenkar

**Affiliations:** 1 School of Zoology, George S. Wise Faculty of Life Sciences, Tel Aviv University, Tel Aviv, Israel; 2 Steinhardt Museum of Natural History, Israel National Center for Biodiversity Studies Tel Aviv University, Tel Aviv, Israel; National Cheng Kung University, TAIWAN

## Abstract

Applying a proteomic approach for biomonitoring marine environments offers a useful tool for identifying organisms’ stress responses, with benthic filter-feeders being ideal candidates for this practice. Here, we investigated the proteomic profile of two solitary ascidians (Chordata, Ascidiacea): *Microcosmus exasperatus*, collected from five sites along the Mediterranean coast of Israel; and *Polycarpa mytiligera* collected from four sites along the Red Sea coast. 193 and 13 proteins in *M*. *exasperatus* and *P*. *mytiligera*, respectively, demonstrated a significant differential expression. Significant differences were found between the proteomes from the northern and the southern sites along both the Mediterranean and the Red Sea coasts. Some of the significant proteins had previously been shown to be affected by environmental stressors, and thus have the potential to be further developed as biomarkers. Obtaining a proteomic profile of field-collected ascidians provides a useful tool for the early-detection of a stress response in ascidians worldwide.

## Introduction

Marine environments, and coastal regions in particular, are heavily exposed to anthropogenic stressors. These fragile habitats are affected by vigorous development, in the form of ports and factories, as well as by tourism and leisure enterprises. Other factors of disturbance result from land-based contaminants reaching the sea via rivers and floods [1–3]. There are various tools currently in use by which to monitor the health of marine ecosystems. Routine chemical assessment is a common practice worldwide; however, it provides a limited ‘snapshot’ of the specific time point of measurement. In addition, chemicals are not always identified nor are they concentrated at a level that enables detection. Thus, sporadic events, such as sewage spills, may easily be missed. Furthermore, chemical analysis does not provide information concerning the effect on living biota [4,5]. Another tool used for quality assessment is biocenotic indexes, i.e. the presence or absence of indicator species within a community, and their tolerance to pollutants. However, this tool is limited to use for well-studied and defined communities for which there are sufficient data available. It is not able to provide an early warning of disturbance [4,6].

Proteins are the immediate effectors in biological processes and provide an accurate account of the current state of a cell, tissue, or the entire organism. In many cases the response to a change in environmental conditions will be reflected in the response of a protein network, independent of gene expression or translation of new mRNA transcripts. Thus, proteins serve as excellent biomarkers directly linking impact and physiological response [4,7]. Proteomics-based approaches are capable of illuminating protein differential expression or alterations without previous knowledge of toxicity mechanisms, although a profound knowledge of the mechanism of action of an enzyme array, for instance, may be necessary [4,8,9]. Applying ‘shotgun’ proteomics (also termed ‘bottom-up’ proteomics), a method in which peptide fragments are correlated to protein sequences in the database [10], enables the identification of proteins or their homologues, pointing at possible expression pathways as a result of a changed environment, and potential new protein biomarkers. This emerging field of environmental proteomics further allows for the integration of non-model organisms that comprise the majority of the environment and are the most ecologically relevant [9,11,12]. Shotgun proteomics has been applied in studies focusing on the response of mussels to pharmaceuticals [13], crustaceans to heavy metals [14] and oysters to ocean acidification [15].

Ascidians (Chordata, Ascidiacea) are sessile filter-feeding organisms closely related to vertebrates [16], able to filter even minute particulate matter [17,18]. They are well known for their ability to accumulate heavy metals [19,20] and to thrive in eutrophic (nutrient-rich), and polluted environments [21]. There are nearly 3000 species of ascidians inhabiting all marine habitats from shallow water to the deep sea, with some species, in particular invasive species, creating large aggregations on artificial structures [21,22]. Their ability to bioaccumulate a variety of contaminants and tolerate large fluctuations in salinity, temperature, and even pollution [23], make them ideal candidates for bio-monitoring a wide variety of marine habitats on a global scale, and for investigating the pathological effects of a variety of stressors. In addition, annotated genome sequences are available for key ascidian species such as *Ciona intestinalis* [24] and *Botryllus schlosseri* [25], further facilitating the use of changes in gene expression in environmental toxicology. The current study focuses on the identification of proteins that could serve as biomarkers of a stress response, with emphasis on the differentially-expressed proteins that may play a role in cellular stress-response mechanisms. Our analysis enabled us to identify a large number of significantly differentiated proteins expressed in the studied species, some of which are known to be stress-related, that could be further established as biomarkers and used for assessing the health of coastal marine environments.

## Materials and methods

### Ethics statement

This study was carried out in strict accordance with the Israeli law. The Israeli Nature and National Parks Protection Authority provided permission to collect the animals and work at all field locations (permit 2017/41626).

### Ascidians and sampling sites

**Fig 1:**
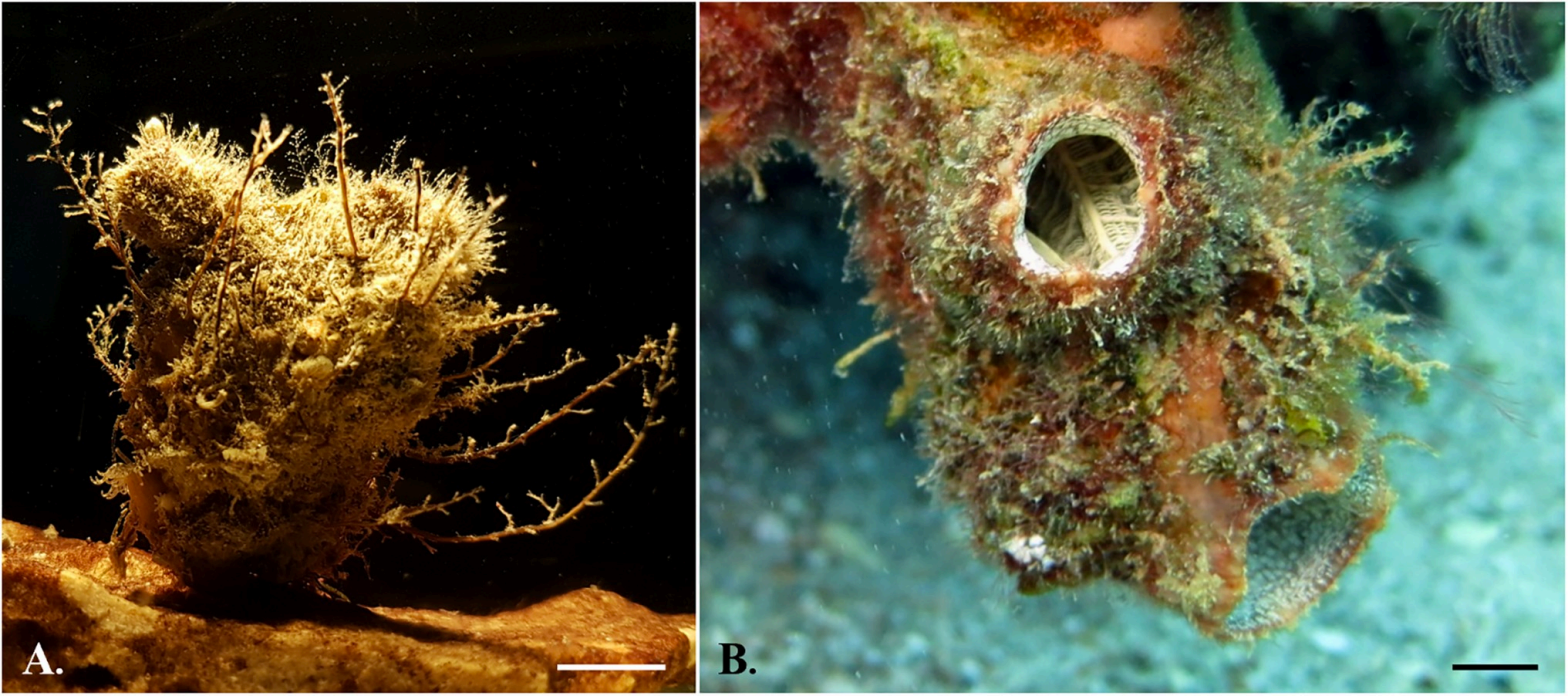
Study species. The solitary ascidians *M*. *exasperatus* (A) and *P*. *mytiligera* (B), sampled from the Mediterranean and from the Red Sea, respectively. Scale bar ~ 1 cm. Photographs by Z. Kuplik (A) and G. Koplovitz (B).

**Fig 2:**
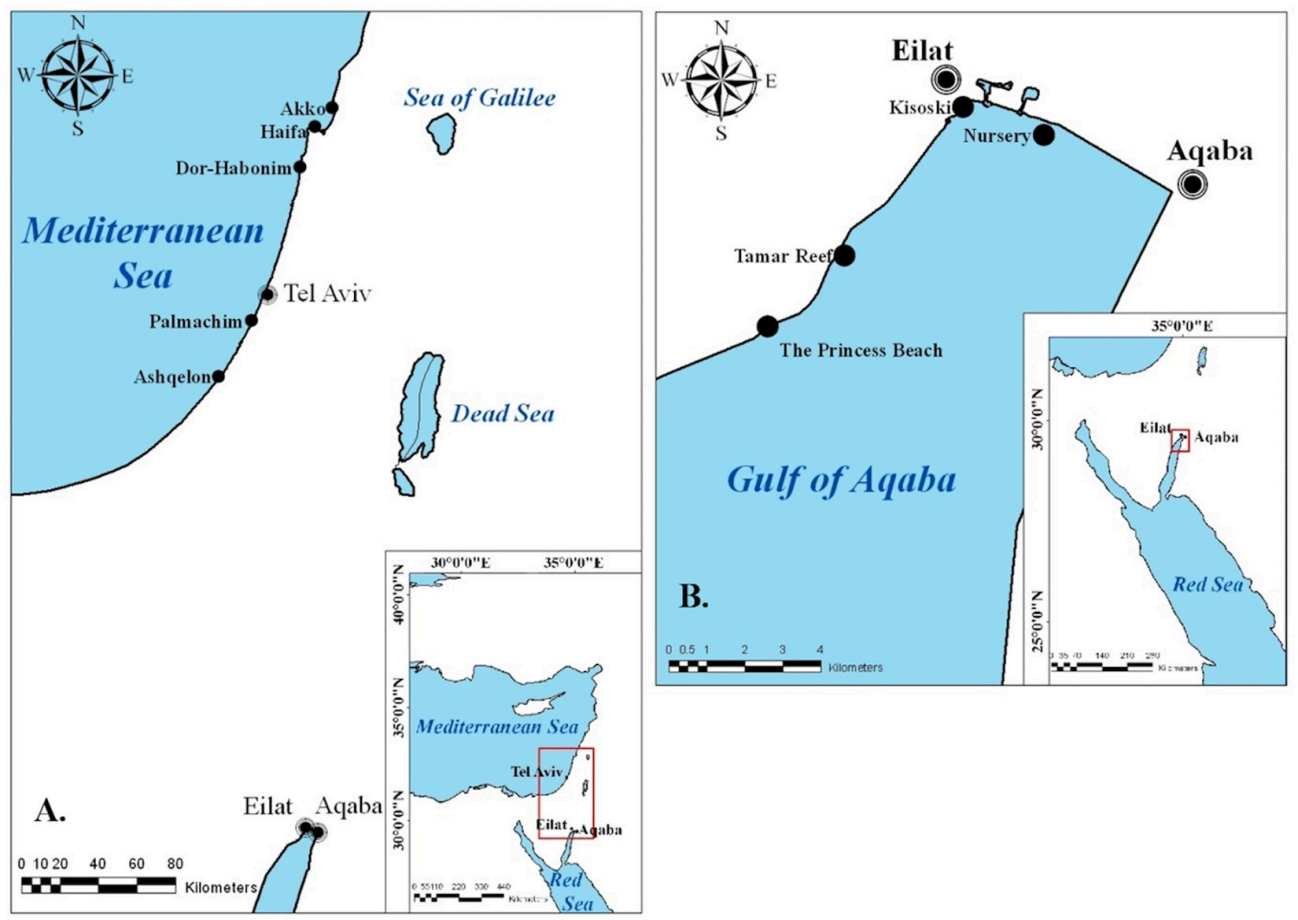
Study area and location of sampling sites. (A) The Mediterranean coast of Israel and (B) the Red Sea. Coordinates are provided in Table 1.

*Microcosmus exasperatus* Heller 1878 (Stolidobranchia; Pyuridae) (Fig 1A) has a wide global distribution and is very common in tropical and sub-tropical waters. It is considered a non-indigenous species to the Eastern Mediterranean, commonly found along the Israeli Mediterranean coast on both artificial and natural substrates [26,27], known to reproduce in this area year-round, except for the winter months (January-February) [28]. This species was sampled from five sites along the Mediterranean coast of Israel (Fig 2A), representing different levels of disturbance: The two northern sites, Akko and Haifa, are known as the most perturbed sites due to years of exposure to various pollutants from multiple sources and due to their proximity to Haifa Port. Dor-Habonim is considered as the least disturbed site and was previously selected as a control site in the National Monitoring Program of Israel’s Mediterranean waters [29]. The two southern sites, Palmachim and Ashqelon, are also known as disturbed sites due to being exposed to episodic pollution events from wastewater and sewage; the Dan Region Wastewater Treatment Plant (Shafdan), Soreq River estuary, and sewage spills from Gaza. Ascidians in Palmachim and Ashqelon were collected from natural reefs and in Akko, Haifa, and Dor-Habonim from patches of rocks scattered on the seabed. Due to technical limitations, sampling of *M*. *exasperatus* from Haifa site was executed a month later than from other sites (June vs. July) and as a consequence the temperature in Haifa at time of sampling was higher (Table 1).

**Table 1:** Sampling data of *M*. *exasperatus* and *P*. *mytiligera* collected for protein profiling. n = 15 (per site). SW = seawater.

Species and location	Date	Site	Coordinates	Depth (m)	SW temperature (°C) *
*M*. *exasperatus* (Mediterranean Sea)	01/06/2017	Akko	32°55'15.0"N, 35° 4'27.0"E	1–3	22.8
	18/07/2017	Haifa	32°49'58.0"N, 34°59'26.0"E	2–3	29.8
	02/06/2017	Dor-Habonim	32°38'40.0"N, 34°55'26.5"E	1–3	23.3
	08/06/2017	Palmachim	31°55'39.0"N, 34°41'38.0"E	4–7	23.6
	08/06/2017	Ashqelon	31°39'57.0"N, 34°32'31.0"E	5–8	23.6
*P*. *mytiligera* (Red Sea)	20/6/2017	The Princess Beach	29°29'43.3"N, 34°54'28.1"E	5	24.8
	20/6/2017	Tamar Reef	29°30'44.2"N, 34°55'34.0"E	6–7	24.8
	19/6/2017	The Coral Nursery	29°32'27.0"N, 34°58'24.0"E	13–15	24.9
	19/6/2017	Kisoski	29°32'50.5"N, 34°57'15.4"N	13–15	24.9

* For the Mediterranean sampling sites, seawater temperature data were derived from the Hadera IOLR (Israel Oceanographic and Limnological Research) monitoring station (sensor at 12 m depth, mean of maximum and minimum recorded temperatures), while for The Red Sea sampling sites temperature data were derived from the IUI (The Inter-University Institute for Marine Sciences) monitoring station (sensor at 1 m depth, temperature recorded at 12 pm).

*Polycarpa mytiligera* (Stolidobranchia; Styelidae) (Fig 1B) is an abundant solitary ascidian native to the Red Sea [30,31]. Similar to *M*. *exasperatus*, it reproduces nearly year-round, excluding the coldest winter months (January-February) [32] It was collected from four different sites along the Israeli coastal stretch of the Red Sea, near the city of Eilat (Fig 2B). Each of these sites is characterized by different water conditions, current regime, and proximity to terrestrial runoff. The two northern sites in the Red Sea, the Nursery and Kisoski, are devoid of a natural coral reef and are located in close proximity to the city of Eilat; whereas the southern sites, Tamar Reef and Princess Beach, are part of a coral-reef marine reserve, with Tamar Reef being an artificial underwater construction. Unlike the other three sites, the Nursery site is a floating structure in the water column, 10 meters above the sea floor. In addition, the northern coasts and sites of Eilat have been exposed to multiple eutrophication events over the years [33,34]. Located at the site of the former fish cages, which had a major impact on nutrient enrichment [34,35], the Nursery site is exposed to freshwater runoff, especially at times of flooding, and is located in proximity to an open canal (the “Kinet” canal) that carries brackish water and terrestrial solvents into the sea [33].

At each site, 15 animals were sampled by SCUBA or snorkeling at depths ranging from 1 to 15 m. Sampling data are given in Table 1 and Fig 2.

Both species were detached from the substrate with utmost care by hand or, when the ascidian was attached to a crevice, using a knife. However, whereas detaching *M*. *exasperatus* from the substrate was fairly easy to perform, detaching *P*. *mytiligera* was more difficult.

Upon removal from the water, all individuals were transferred within a few minutes to liquid nitrogen, in order to minimize a biased protein expression profile. As a general rule, batches of three individuals were wrapped together in aluminum foil prior to freezing, since direct flash-freeze had been shown to result in shattered specimens. Samples were transferred frozen to the laboratory at Tel Aviv University, either on dry-ice or in liquid nitrogen, where they were kept at -80 °C until processed. At the lab, the tunic of each animal was removed on ice for minimal thawing, and the inner bodies of every three ascidians were pooled into one sample. Removing the tunics was essential since the tunics of both species were covered with epibionts which could have affected the protein expression profile. When specimens were found shattered, making it difficult to distinguish between individuals, their fragments were jointly weighed and divided into equal weight samples. In total, each site produced five replicas (i.e. N = 5) of three individuals.

### Protein extraction

All protein-related procedures were performed at the Smoler Proteomics Center, Technion, Haifa. Tissue was homogenized at a ratio of 1 g tissue: 3 mL buffer, composed of: 8 M Urea, 100 mM Ammonium bicarbonate, 10 mM DTT. Prior to digestion, 2 mL homogenates were sonicated (to break up any remaining tissue chunks) and briefly centrifuged to pellet insoluble debris. Protein amount was estimated using Bradford protein assay. 20 g of protein from each sample were reduced with 10 mM DTT (60 °C for 30 min.) and modified with 8.8 mM iodoacetamide in 400 mM ammonium bicarbonate (in the dark, at room temperature for 30 min.), followed by digestion overnight at 37 °C in 2 M Urea, 25 mM ammonium bicarbonate with modified trypsin (Promega) at a 1:50 enzyme-to-substrate ratio. A second digestion with trypsin was done for 4 hours at 37 °C. Protein extraction and trypsinization of the different samples were done at the same day to reduce technical variability.

### Protein analysis by LC-MS/MS

The tryptic peptides were desalted using C18 tips (Harvard), dried and then re-suspended in 0.1% Formic acid. Two μg of the resulted peptides were resolved by reverse-phase chroμatography on 0.075 X 180-mm fused silica capillaries (J&W) packed with Reprosil reversed phase material (Dr Maisch GmbH, Germany). The peptides were eluted with a linear gradient of acetonitrile in 0.1% formic acid in water, as detailed: 5 to 28% for 180 min., 28 to 95% for 15 min., and 95% acetonitrile for 25 min. Flow rate was 0.15 μL/min. Mass spectrometry was performed by Q Exactive plus mass spectrometer (Thermo Scientific) in a positive mode (m/z 350–1800, resolution 70,000) using repetitively full MS scan followed by collision induces dissociation (HCD) of the ten most dominant ions (> 1 charges) selected from the first MS scan. A dynamic exclusion list was enabled with exclusion duration of 20 sec. LC-MS/MS analyses of the different samples were done as one set on the same week with washes and empty runs between them to avoid contaminations.

The MS raw data from all the biological repeats were analyzed using the MaxQuant software (version 1.5.2.8) for peak picking and quantitation, followed by identification using the Andromeda search engine vs. the Ascidiacea database- (Taxon ID 7713, 56, 748 proteins) and *Ciona intestinalis* subsets of the uniprot database, and against Stolidobranchia subset of NCBI database, with mass tolerance of 20 ppm for the precursor masses and fragment ions. Methionine oxidation was set as variable post-translational modifications, and carbamidomethyl on cysteine as a static one. Minimal peptide length was set to six amino acids, and a maximum of two miscleavages was allowed. To eliminate identifications from the reverse database and common contaminants, peptide- and protein-level false discovery rates (FDRs) were filtered to 1% using the target–decoy strategy. Data were quantified by label free analysis using the same MaxQuant software, based on extracted ion currents (XICs) of peptides from each LC/MS run, enabling quantitation of all the peptides identified in any experiment. Normalization was done using the LFQ algorithm base on similar protein profile [36]. Since several organisms were included in the database, homolog proteins were clustered as one protein group, and only proteins that were identified with at least two peptides were listed. As the data need to be in a linear scale, in order to apply the statistical tests, they were transformed to log2 intensities.

### Statistical analysis

Significant differences in protein expressions between sites of each sea were tested via two-sample *t*-tests with Permutation-based FDR (with 250 randomizations, threshold value = 0.05).

Additionally, hierarchical clustering-based heatmaps were produced in order to easily identify up or down-regulated proteins. Both analyses were performed with Perseus 1.6.0.7 software (Mathias Mann’s group). Only proteins that met a minimum of 60% valid value criteria (in our study three out of five repeats were needed to provide an intensity reading) were used for the analyses.

In order to reveal any grouping of sites, a principal component analysis (PCA) of significantly expressed protein profiles was undertaken, followed by analysis of similarity (ANOSIM). A similarity percentages analysis (SIMPER) was used to indicate percentage of similarity/dissimilarity between sites and to identify the proteins whose expression contributed the most to the observed dissimilarities. PCA, ANOSIM and SIMPER were performed with the PRIMER-E software, version 6. Significant differences were accepted when *p-*values were < 0.05.

## Results and discussion

### Identified proteins and biological functions

**Fig 3:**
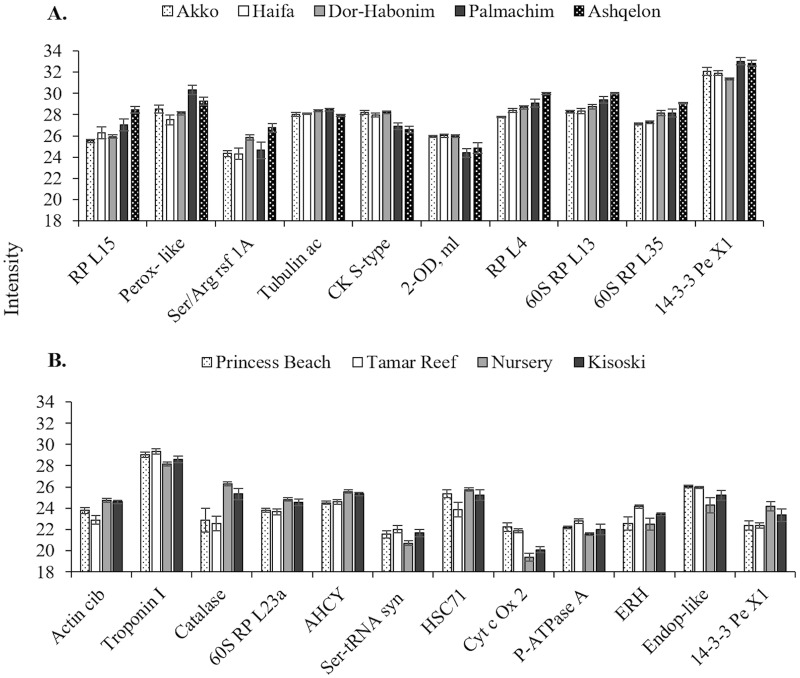
Comparison of the most pronounced proteins that show significant differential expression between sampling sites. (A) of the Mediterranean, and (B) of the Red Sea. Protein abbreviations are given in S1 and S2 Tables. For (A): Ak = Akko, Ha = Haifa, DH = Dor-Habonim, Pa = Palmachim, As = Ashqelon. For (B): PB = Princess Beach, TR = Tamar Reef, NSY = The Nursery, KSI = Kisoski. For both (A) and (B) n = 5, ±SE.

**Fig 4:**
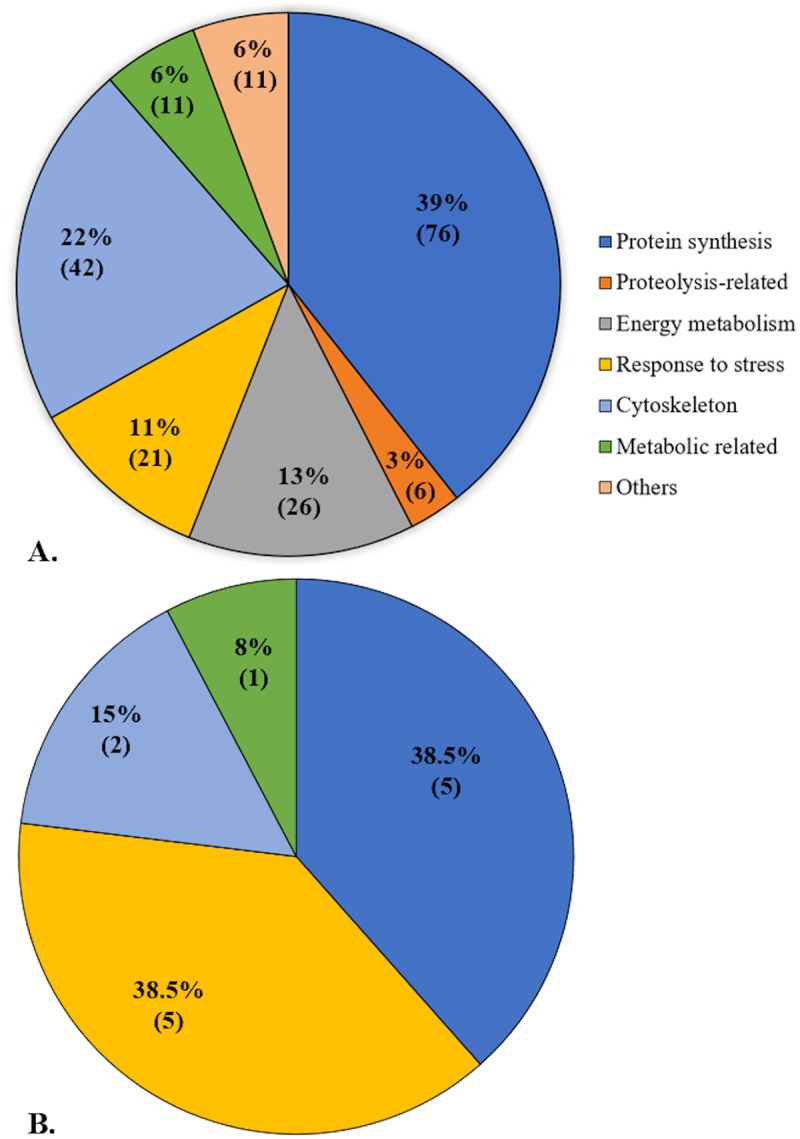
Biological functions associated with significantly differentially expressed proteins in the sampled ascidians. (A) *M*. *exasperatus*, and (B) *P*. *mytiligera*. Percentage and number of proteins are indicated for each category.

Overall, 431 proteins were identified from *M*. *exasperatus* sampled at the Mediterranean sites, of which 403 had met the 60% minimum valid value criteria and were used for analyses. Of these, 193 proteins showed significant differential expression between sampling sites (*p* < 0.05, two-sample *t*-test) (Fig 3A; for a complete list of significant proteins see S1 Table and S1 File) and were assigned to several main functional categories: protein synthesis, cytoskeleton, energy metabolism, response to stress, metabolic-related and proteolysis-related (Fig 4A). Over 60% of the significant proteins were associated with protein synthesis (39%) and cytoskeleton (22%). Since data on the proteome of ascidians in the field are scarce, this finding (i.e. the plethora of proteins with which to experiment) is of high importance. In addition to proteins such as peroxiredoxin, hsp70 and 14-3-3, all of which are stress-related and were previously suggested to serve as universal indicators for cellular stress [37,38], other proteins associated with various biological functions may also qualify as biomarkers. For example, the synthesis of ribosomal proteins, which comprised most of the proteome in our study, is affected by the cell’s environment, repressed in response to stress, and induced under favorable conditions [39]. In addition to their role in ribosome biogenesis and cellular development [40], many ribosomal proteins have critical roles in various cellular functions, such as DNA repair [41], apoptosis [42], and cellular differentiation [43]. Due to their relatively high abundance in the proteome of the sampled *M*. *exasperatus*, correlating changes in ribosomal proteins expression to various environmental factors/stressors could establish them as valuable biomarkers.

In contrast to the large number of identified proteins from *M*. *exasperatus*, only 126 were identified from *P*. *mytiligera* individuals sampled at the Red Sea. Of the 70 proteins that met the 60% minimum valid value criteria, 13 proteins significantly differentiated exclusively between the two southern sites (Tamar artificial reef and the natural reef of Princess Beach) and the northern unique site (Nursery) in the northern zone (*p* < 0.05, two-sample *t*-test) (Fig 3B, S2 Table and S2 File). These proteins were assigned to four main functional categories (response to stress, protein synthesis, cytoskeleton, and metabolic-related), with most proteins (77%) associated with the former two categories, five proteins in each (Fig 4B). Although these species belong to different families (Pyuridae and Styelidae), they are of the same order (Stolidobranchia) and the same database was used for identification (Ascidiacea). The obtained results are therefore surprising, since fewer proteins were identified for *P*. *mytiligera*. However, it should be noted that because the animals were sampled from different habitats and under different conditions, we do not expect to obtain the same results. Moreover, the additional effort required to detach *P*. *mytiligera* from its substrate may also account for a different pattern of protein expression. The list of significant proteins nonetheless includes potential biomarkers for various stressors such as actin [44], 14-3-3 proteins [37], and Hsc71 [45].

### Distinction between sites/zones

**Fig 5:**
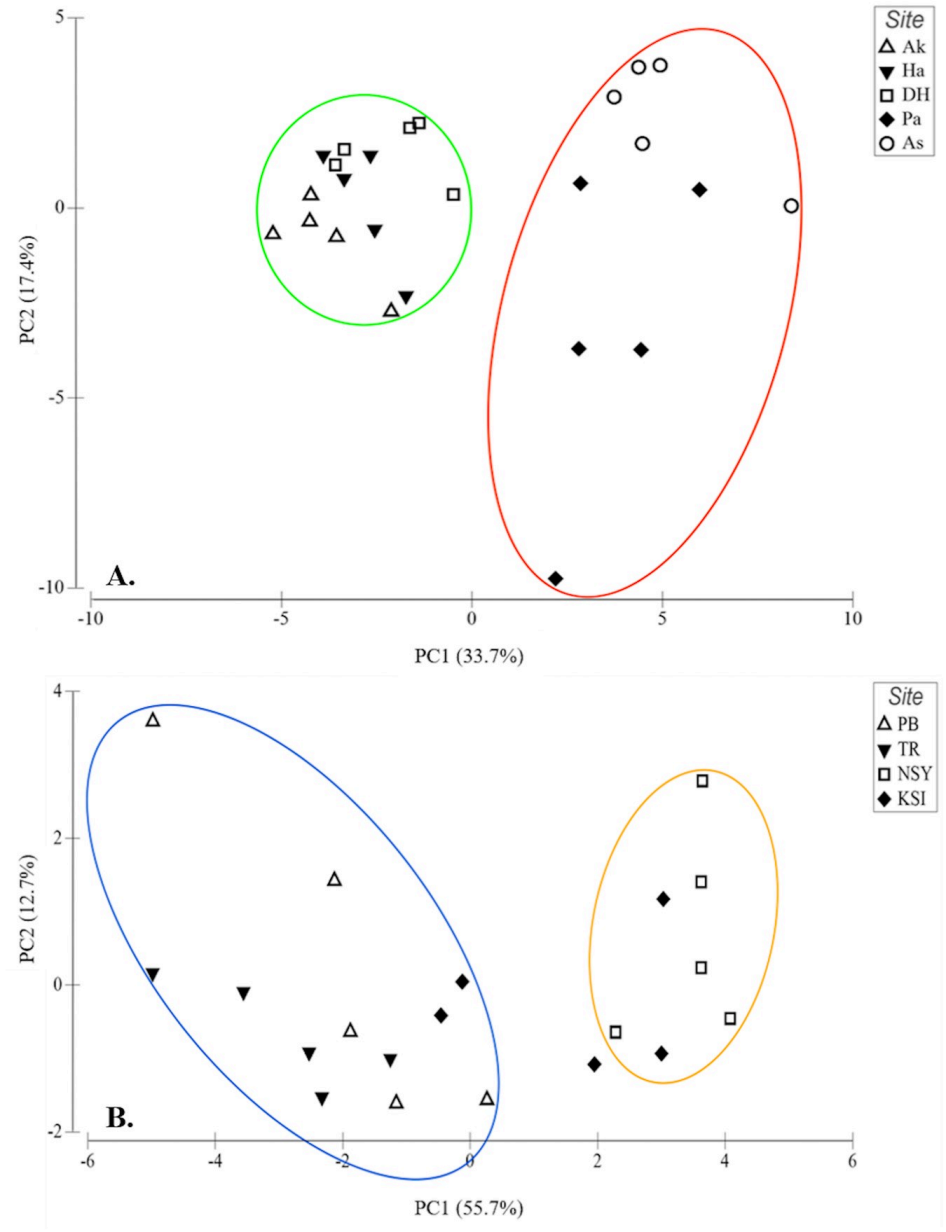
PCA of sampling sites for *M*. *exasperatus* (A) and *P*. *mytiligera* (B). Only proteins that showed significant differential expression among sites were used for the analysis; 193 for Mediterranean sites and 13 proteins for the Red Sea (*p* < 0.05; two-sample *t-*tests with permutations). Encircled are groupings of sites found to be significantly dissimilar. For (A): Ak = Akko, Ha = Haifa, DH = Dor-Habonim, Pa = Palmachim, As = Ashqelon. For (B): PB = Princess Beach, TR = Tamar Reef, NSY = Nursery, KSI = Kisoski.

**Fig 6:**
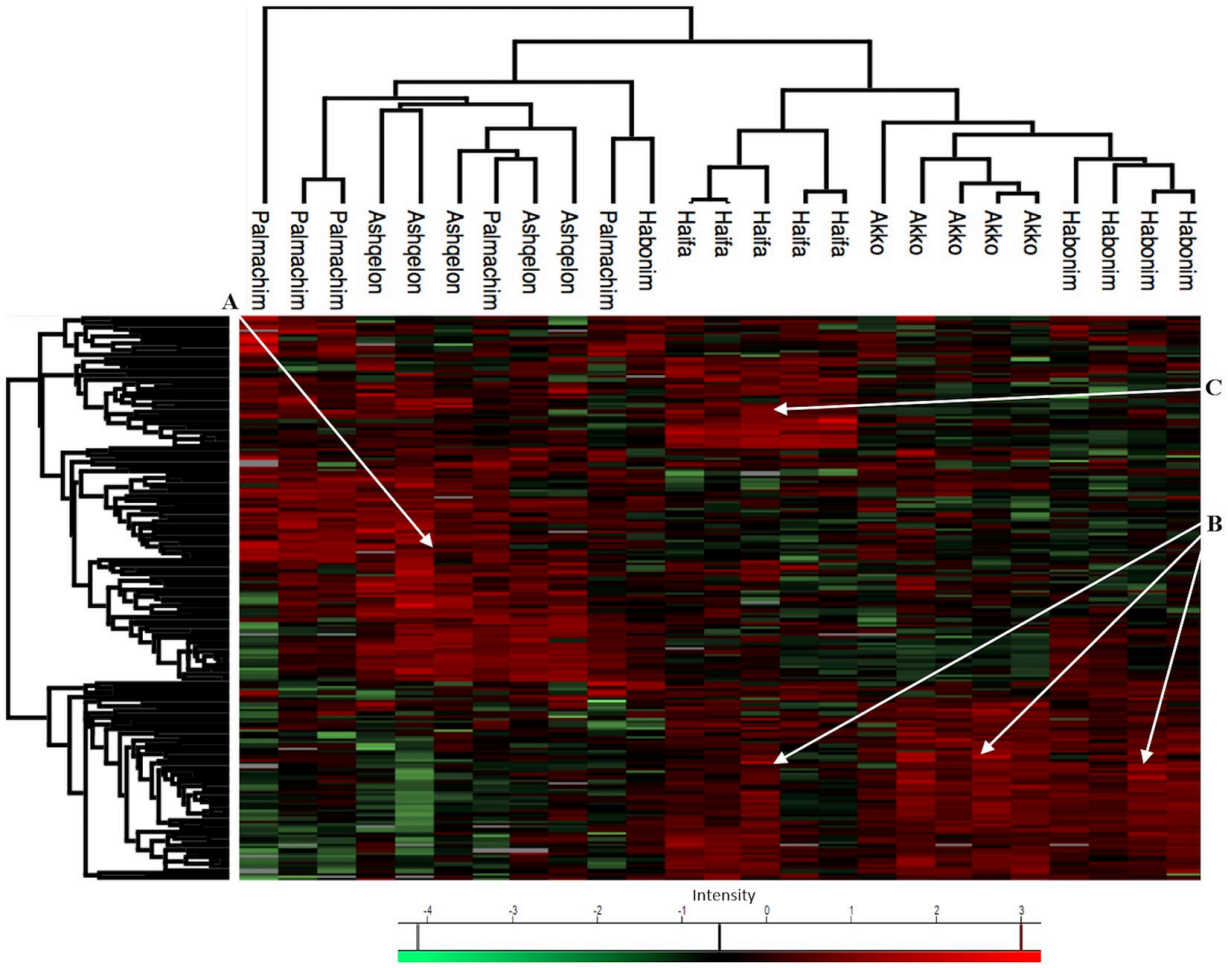
Hierarchical clustering-based heatmap of significantly differentiated proteins expressed in *M*. *exasperatus*. Up-regulated protein clusters of specific zones and sites are indicated by arrows: For the southern sites (A), for the northern sites (B), for Haifa (C).

PCA (Fig 5A), and hierarchical clustering-based heatmap (Fig 6) of the significant proteins showed a clear separation between northern (Akko, Haifa and Dor-Habonim) and southern sites (Palmachim and Ashqelon) of the Mediterranean.

**Fig 7:**
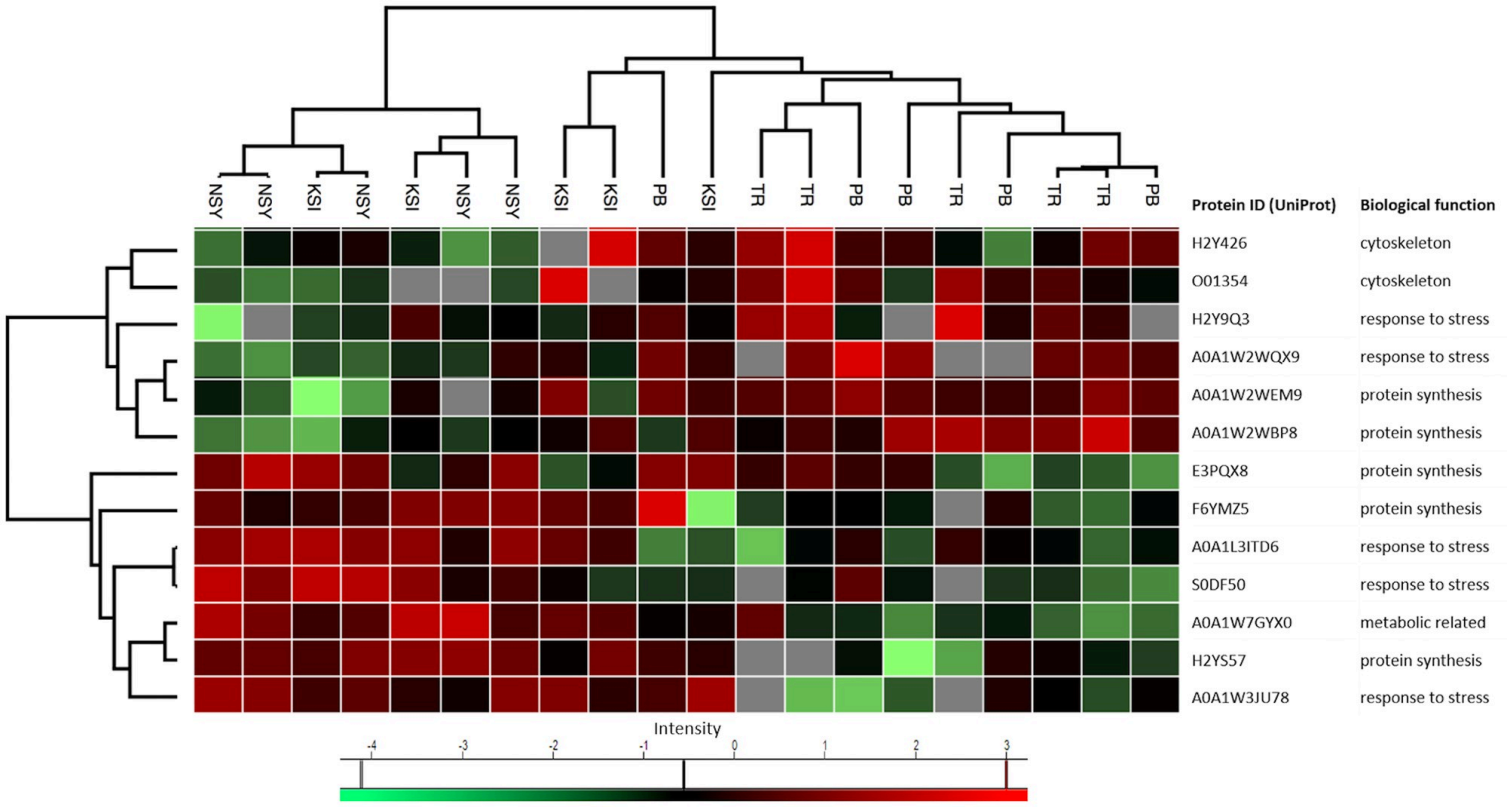
Hierarchical clustering-based heatmap of significantly differentiated proteins expressed in *P*. *mytiligera*. PB = Princess Beach, TR = Tamar Reef, NSY = Nursery, KSI = Kisoski.

Most apparent were clusters of upregulated proteins, with 68 upregulated proteins in the northern sites and 80 in the southern sites. The majority of proteins in the northern sites were cytoskeletal proteins (26, 38%) and energy metabolism related (22, 32%), whereas most proteins in the southern sites were assigned to protein synthesis (53, 66%). ANOSIM for the two zones (i.e. north vs. south), confirmed that they were significantly dissimilar (R = 0.868, *p* = 0.001). ANOSIM for sites (global R = 0.731, *p* < 0.01) showed that the most dissimilar sites were Akko and Ashqelon (pairwise test R = 1, *p* = 0.008). Although less apparent from the PCA, ANOSIM revealed significant dissimilarities among northern sites too, with Haifa being most dissimilar to the other two sites; Akko and Haifa (R = 0.836, *p* = 0.008), Haifa and Dor-Habonim (R = 0.904, *p* = 0.008) and Akko and Dor-Habonim (R = 0.616, *p* = 0.008). This difference among northern sites was more noticeable in the heatmap, where a cluster of 31 upregulated proteins distinguished Haifa from Akko and Dor-Habonim (S1 Fig). A distinction between zones/sites was demonstrated for *P*. *mytiligera* too, with noticeable differences between the southern sites and the Nursery site in the north (Fig 5B). This can be seen also in the hierarchical clustering-based heatmap (Fig 7), in which roughly two clusters of proteins differentially up-regulated between the northern Nursery site and the two southern sites. Although *t*-test results indicate that expression of proteins at Kisoski was not significant, ANOSIM (global R = 0.527, *p* < 0.01) suggests that protein profiles of organisms sampled at this site were slightly dissimilar to those sampled at Princess Beach and Tamar Reef (southern sites, R = 0.376, *p* = 0.016 and R = 0.62, *p* = 0.008, respectively).

Seven proteins were up-regulated at the Nursery site vs the two southern sites, belonging to the following categories: Cytoskeleton (1, 14%), response to stress (3, 43%), and protein synthesis (3, 43%). SIMPER analysis shows that the proteins which contributed most to the observed differences between the southern sites and the Nursery were catalase (17.8%) and cytochrome c oxidase subunit 2 (13.2%), both response to stress proteins.

The observed dissimilarities between sites and zones in both seas are most intriguing. Although Dor-Habonim is considered a relatively clean site and was previously selected as a control site in the National Monitoring Program of Israel’s Mediterranean waters [29], the PCA indicated that protein profiles of *M*. *exasperatus* sampled at this site are closely related to those of Akko and Haifa. Since these sites are located in Haifa Bay, a region which has been polluted by a variety of contaminants for many years, greater dissimilarity between clean and polluted sites would be expected. The present finding, however, indicates that other factors may be involved: e.g. food availability, as well as specific combinations of hydrological conditions and various pollutants [46,47], could have masked the effect of contaminants on organisms from Haifa Bay and have resulted in what seems to be a mismatch. Further investigation of the complex of conditions at each site is required in order to better elucidate these results. Nevertheless, although subtle, significant differences were observed among the northern sites, with Haifa being most dissimilar to the others. A possible explanation for the dissimilarity between Haifa and the two other northern sites can be found in the large proportion of stress-related proteins within the cluster of 31 upregulated proteins (14, 45%), including hsp90s alpha and beta, hsp70 and endoplasmin-like. Heat-shock proteins, which are known to be very important to the survival of cells and organisms in response to various stressors [48], may suggest that organisms of this habitat may be exposed to different or additional stressors than those at Akko and Dor-Habonim. Interestingly, since sediments in both Akko and Haifa have been reported as intermediately to heavily polluted by various contaminants such as toxic organic substances (e.g. PCB’s) and heavy metals [49], it should be expected that sedentary organisms residing in both habitats and exposed to similar pollutants would exhibit similar protein profiles. The observed differences between sites could be the result of expansion of the Haifa Port project which has been operating for the last couple of years, and the extensive dredging works involved. Due to the proximity of the Haifa site to the port, organisms in this habitat may be exposed to an increased suspended sediment load, and hence to higher concentrations of contaminants. A possible bias in our results may be due to the temperature difference between Haifa and the other sites: since seawater temperature at Haifa was 6 °C higher than that at the other sites at the time of sampling (Table 1), expression levels of some proteins, such as hsp70, could have been affected by the temperature difference. However, since not all proteins are temperature-dependent and not all members of the hsp family are affected by natural temperature fluctuations (e.g. hsp90 [50]), we suggest that the observed differences were probably associated with the heavy pollution and other perturbations to which organisms at this site have been continuously exposed.

With respect to the clear differences between protein profiles of ascidians from the northern and southern sites in both the Mediterranean and the Red Sea, several possible explanations exist: first and foremost, the special feature of the Nursery site in the Red Sea, floating in the water column high above the seabed. This is a probable cause of the observed differences between the proteome of *P*. *mytiligera* from this site and those from Princess Beach and Tamar Reef. Environmental factors, including mechanical disturbance, have been found to impact cell metabolism and also the expression of stress proteins such as hsp70 [51,52]. The investment of less energy by ascidians in removing suspended sediment matter at the Nursery site could have resulted in the observed differences between sites. This may also explain the differences between the protein profiles of Dor-Habonim and the two southern sites of the Mediterranean: whereas *M*. *exasperatus* in Dor-Habonim was collected from substrate in close proximity to the sediment, the ascidians in Palmachim and Ashqelon were sampled from natural reefs, considerably higher above the sediment.

The lack of a natural coral reef at the northern sites of the Red Sea, unlike the southern sites, which are located within a coral-reef marine reserve, is reflected also in their sediment: in the northern sites, sediment particles are smaller and of terrestrial origin, while sediment at the southern sites is composed of larger particles of marine biogenic origin [33]. Although the northern sites have experienced nutrient enrichment events during past years, the findings of the national monitoring program did not reveal any contaminants as such (e.g. terrestrial runoff) in the year preceding our sampling [33]. Thus, the observed differences in protein expression between the northern and southern sites of the Red Sea could not be attributed to such events.

In regard to the Mediterranean sites, although both northern (Akko and Haifa) and southern (Palmachim and Ashqelon) sites are subjected to various pollutants, protein profiles were significantly different. It is reasonable to assume that this finding reflects the differences between the substances the ascidians are exposed to in the two regions, as well as the pattern of exposure. Whereas *M*. *exasperatus* in the Bay of Haifa (i.e. Akko and Haifa sites) are chronically exposed to a large number of toxicants, as this area is in close proximity to the largest petrochemical plants in Israel, as well as to one of its largest ports, ascidians in Palmachim and Ashqelon are exposed intermittently to pollutants from other sources, mostly from wastewater and sewage.

Although significant, SIMPER analysis indicated that the observed dissimilarities were not greater than 1.1% in the Mediterranean and 3.2% in the Red Sea. We suggest that this is due to our processing and analyzing whole organisms rather than selected tissues. As was previously shown, the expression profile and magnitude of specific proteins in response to known contaminants may differ between tissues. Lopez et al.’s [53] study on the proteomic response to elevated temperatures in ovaries of the solitary ascidian *Ciona intestinalis*, revealed that many of the identified proteins were stress-related. Chora et al. [54] showed that actin, an important cytoskeletal protein, was expressed differently in the gills and digestive glands of the clam *Ruditapes decussatus* when exposed to cadmium. It is probable that if we had analyzed selected tissues of the studied ascidians, the relative expression of specific proteins would have been different and dissimilarities would have been more pronounced.

Discussing the obtained results would not be complete without considering possible confounding factors that could have affected the presented protein profiles. In mussels, for instance, factors such as age, reproduction stage, and food availability were shown to induce various biological responses that could be misinterpreted as an exposure effect [55,56,57]. Similarly, in the present study, the obtained site-specific differences in protein expression levels could not be linked to a specific stressor, endogenic or exogenic. Nevertheless, considering the differences in pollution levels that the sampling sites are exposed to and the major sources of the pollutants (i.e. anthropogenic sources), these findings demonstrate the potential of ascidians to function as bioindicators, possibly of anthropogenic stressors.

That being said, since the combined effect of various stressors on ascidians in their natural environment has not been investigated to date, the subject merits further study. Isolated populations (e.g. due to geographical distance) could also have led to biased conclusions, with site-specific selection pressures possibly yielding differences in protein profiles. Nevertheless, both species used in this study are solitary ascidians that spawn their gametes into the water column. As the gametes may spend a period of hours to several days in the water until fertilization, and the developing embryos also drift in the water for several hours, the distance from release of gametes to settlement of the larvae may reach hundreds of kilometers. A similar pattern of dispersal was previously demonstrated for *Pyura gibbosa gibbosa* [58], a solitary ascidian of the same family as *M*. *exasperatus* (Pyuridae), for which five populations of *P*. *gibbosa gibbosa* located in a distance of up to 215 kilometers apart from each other were found to support a single panmictic population. Considering the limited geographical distance between the sampling sites in our study, 147 km and 17 km, Mediterranean and Red Sea respectively, and a dominant south-to-north current in the Mediterranean (similar to the unidirectional current that exists in Ayre et al.’s (1997) study region, which was assumed to link between sampling sites), it is unlikely that either of the species has established isolated populations within the limited geographic distribution. A future population genetic study should clarify whether these populations are panmictic or reproductively isolated.

## Conclusions

In the current study, we demonstrate how ascidians from different localities exhibited significantly differentiated protein profiles, which could reflect the conditions of the environments they inhabit. The obtained proteomes distinguished between sites, providing essential information regarding the current environmental health status at these areas, thus providing us with a more accurate estimation of the health of a specific site. Our results show evidence for differentially-expressed proteins, which are stress-related or previously suggested to serve as universal biomarkers in other studies. We find the large number of identified differentiated proteins which we obtained advantageous for an additional reason: Due to the complexity of pollutants in the environment and the wide spectrum of proteomic responses these may induce in organisms [7], a combination of well-studied proteomic biomarkers, rather than a single one, could be more efficient in identifying the stressors involved and would improve the environmental hazard assessment (reviewed by Trapp et al. [4]).

These findings corroborate the potential of the proteomic approach for monitoring coastal marine environment health, with ascidians as bioindicators. Furthermore, the study also presents the first application of ascidians as bioindicators in coral reefs, in regard to the southern sites of the Red Sea. Additional controlled exposure experiments and field studies will contribute to our understanding of the stress-response pathways involved, and will enable validating the potential of proteins of interest to serve as qualified biomarkers.

## Supporting information

S1 Table A complete list of significantly differentiated proteins expressed in *M*. *exasperatus*, sampled from the Mediterranean. When possible, molecular function was specified for uncharacterized proteins for which a name was not specified in UniProt. Abbreviations of the proteins which appear in the column chart (Fig 3A) are specified in parentheses, bold font, following their full name. na = not available.
(DOCX)

S2 Table All significantly differentiated proteins expressed in *P*. *mytiligera*, sampled from the Red Sea. Abbreviations of the proteins which appear in the column chart (Fig 3B) are specified in parentheses, bold font, following their full name.
(DOCX)

S1 Fig Heatmap showing a cluster of 31 upregulated proteins in *M*. *exasperatus* from Haifa. Protein names are listed in S1 Table. na = not available, assigned to proteins for which data on their biological functions could not be obtained.
(TIF)

S1 File The analysis data of all significantly differentiated proteins expressed in *M*. *exasperatus*. Intensity values (LFQ) were produced from raw MS data using MaxQuant software, and significant differences between sites were tested via two-sample *t*-tests, using Perseus software.
(XLSX)

S2 File The analysis data of all significantly differentiated proteins expressed in *P*. *mytiligera*. Intensity values (LFQ) were produced from raw MS data using MaxQuant software, and significant differences between sites were tested via two-sample *t*-tests, using Perseus software.
(XLSX)
