# Supplementary material for: Proteomic profiling of ascidians as a tool for biomonitoring marine environments
Source: PLoS One. 2019 Apr 9;14(4):e0215005. doi: 10.1371/journal.pone.0215005 (PMC6456167; doi:10.1371/journal.pone.0215005)
Supplement: S1 Table — When possible, molecular function was specified for uncharacterized proteins for which a name was not specified in UniProt. Abbreviations of the proteins which appear in the column chart (Fig 3A) are specified in parentheses, bold font, following their full name. na = not available. (DOCX) [file pone.0215005.s001.docx]

| **Protein ID (UniProt)** | **Protein name** | **Biological function** |
| --- | --- | --- |
| H2ZPQ6 | tubulin alpha chain | cytoskeleton |
| H2Z7E0 | transaldolase | metabolic-related |
| A0A1W2VNR1 | arrestin | response to stress |
| A0A1W5BBW6 | NFkB protein isoform X1 | protein synthesis |
| A0A1W2W222 | probable splicing factor 3B subunit 5 | protein synthesis |
| H2YCG8 | uncharacterized protein | na |
| A0A1W2WJ24 | catenin alpha-2 | cytoskeleton |
| A0A1W2VNA5 | beta-catenin | protein synthesis |
| A0A1W5BH56 | spectrin alpha chain, non-erythrocytic 1 | cytoskeleton |
| A0A1W2W363 | angiotensin-converting enzyme | metabolic-related |
| A0A1W2WGX8 | alpha-actinin-2 isoform X4 | cytoskeleton |
| A0A1W5BIH0 | filamin-C isoform X12 | cytoskeleton |
| H2ZPF0 | spectrin beta chain | cytoskeleton |
| H2ZQD6 | uncharacterized protein (dipeptidase activity) | proteolysis-related |
| Q9GV21 | cytochrome b5 | energy metabolism |
| Q1EPR8 | GTP binding protein Rho | protein synthesis |
| A0A1W2WM09 | ras-related protein Rab-11B-like | response to stress |
| A0A1W2WLV6 | dolichyl-diphosphooligosaccharide--protein glycosyltransferase subunit STT3A | protein synthesis |
| A0A1W2W936 | dolichyl-diphosphooligosaccharide--protein glycosyltransferase 48 kDa subunit | protein synthesis |
| A0A1W2WLQ2 | coatomer subunit alpha | protein synthesis |
| A0A1W2WAB3 | C-terminal-binding protein 1 | protein synthesis |
| A0A1W2W8Q8 | hydroxyacyl-coenzyme A dehydrogenase, mitochondrial-like | petabolic-related |
| A0A1W2WHF8 | histone H2A | protein synthesis |
| A0A1L3ITD6 | heat shock cognate 71 kDa protein | response to stress |
| A0A1W2WJJ0 | dnaJ homolog subfamily A member 1-like | response to stress |
| A0A1W2W2Q8 | T-complex protein 1 subunit alpha-like | protein synthesis |
| A0A1W3JGG9 | T-complex protein 1 subunit theta-like | protein synthesis |
| A0A1W2W9G4 | RuvB-like helicase | protein synthesis |
| A0A1W2VNY3 | mitogen-activated protein kinase | response to stress |
| A0A1W5BBS3 | 60 kDa heat shock protein, mitochondrial-like | response to stress |
| H2YRN0 | ATP binding | na |
| A0A1W3JIN0 | coatomer subunit beta | protein synthesis |
| A0A1W2W5U3 | glutathione reductase isoform X1 | response to stress |
| A0A1W2WDT0 | protein transport protein Sec23A | protein synthesis |
| H2ZAT3 | ATP binding | na |
| E3PQU8 | heat shock protein 90kDa alpha (Cytosolic) class B member 1 | response to stress |
| A0A1W3JEH1 | transmembrane emp24 domain-containing protein 4-like | protein synthesis |
| A0A1W2WD99 | 78 kDa glucose-regulated protein-like | response to stress |
| A0A1W2WPS5 | 78 kDa glucose-regulated protein-like | response to stress |
| A0A1W3JR55 | protein disulfide-isomerase | protein synthesis |
| L7Q498 | heat shock protein 90 beta | response to stress |
| H2YHC4 | ATP binding | response to stress |
| A0A1W3JU78 | endoplasmin-like | response to stress |
| Q93147 | heat shock protein 70 | response to stress |
| A0A1W3JCR7 | heat shock protein 90-beta | response to stress |
| B8XX92 | vasa | development-related |
| A0A1W2W7U2 | ELAV-like protein 1-B isoform X3 | protein synthesis |
| H2ZHR1 | uncharacterized protein (Arp2/3 complex-mediated actin nucleation) | cytoskeleton |
| A0A1W2WGA0 | calsequestrin | energy metabolism |
| A0A1W2WLH9 | 116 kDa U5 small nuclear ribonucleoprotein component | protein synthesis |
| A0A1W2WJP5 | transmembrane emp24 domain-containing protein 10-like isoform X2 | protein synthesis |
| H2ZFR1 | T-complex protein 1 subunit delta | protein synthesis |
| A0A1W2W232 | proteasome subunit alpha type | proteolysis-related |
| A0A1W3JGZ5 | protein arginine N-methyltransferase 1-like | response to stress |
| O44340 | L-lactate dehydrogenase | energy metabolism |
| P92206 | troponin C | cytoskeleton |
| Q04757 | body wall muscle protein HR-29 | cytoskeleton |
| A0A1W2W6I3 | fructose-bisphosphate aldolase | energy metabolism |
| Q25472 | actin, muscle-type | cytoskeleton |
| A0A1W2WAV4 | mitochondria-eating protein | response to stress |
| A0A1W5B469 | rootletin isoform X2 | protein synthesis |
| H2YJ51 | elongation factor 1-alpha | protein synthesis |
| A0A1W2WIK7 | 40S ribosomal protein S3 | protein synthesis |
| Q69GT9 | ribosomal protein CEP52 | protein synthesis |
| E3PQX1 | ribosomal protein S13 | protein synthesis |
| O97347 | intermediate filament protein IFB | cytoskeleton |
| Q9GVA2 | intermediate filament protein C | cytoskeleton |
| A0A1W2W7C0 | protein mago nashi homolog | protein synthesis |
| A0A1W2WEC0 | spliceosome RNA helicase DDX39B | protein synthesis |
| E3PQW6 | small nucleolar RNA, C/D box 21 | protein synthesis |
| A0A9Q1 | class II myosin heavy chain | cytoskeleton |
| A0A1W5BMX3 | IQ motif containing GTPase activating protein homologue isoform X4 | protein synthesis |
| E3PQX5 | 40S ribosomal protein S27 | protein synthesis |
| A0A1W3JH16 | septin-2B isoform X4 | cytoskeleton |
| A0A1W5BKT1 | cytohesin-1 | protein synthesis |
| F6TJY9 | 40S ribosomal protein S21 | protein synthesis |
| F6YMZ5 | uncharacterized protein (ribosomal small subunit assembly) | protein synthesis |
| A0A1W5BAW2 | myosin-10 isoform X3 | cytoskeleton |
| A0A1W2W3G1 | FRAS1-related extracellular matrix protein 2 | cell communication |
| A0A1W2WB87 | 14-3-3 protein epsilon-like isoform X1 **(14-3-3 Pe X1)** | response to stress |
| A0A1W2WIG1 | 26S protease regulatory subunit 10B | proteolysis-related |
| E3PQV1 | proteasome (Prosome, macropain) subunit, beta type 1 | proteolysis-related |
| A0A1W2VNK9 | peroxiredoxin-like **(Perox-like)** | response to stress |
| A0A1W2WEZ5 | COP9 signalosome complex subunit 2-like | development-related |
| A0A1W3JJG4 | calcium-binding protein 39 | metabolic-related |
| A0A1W2WA45 | eukaryotic peptide chain release factor subunit 1 | protein synthesis |
| H2Y7P0 | uncharacterized protein | na |
| A0A1W2WPG5 | 60S ribosomal protein L11-like | protein synthesis |
| A0A1W2WEW7 | 40S ribosomal protein S5 | protein synthesis |
| A0A1W2WFP3 | elongation factor 1-alpha | protein synthesis |
| A0A1W2WI70 | 60S ribosomal protein L12 | protein synthesis |
| E3PQW9 | 60S acidic ribosomal protein P0 | protein synthesis |
| E3PQX7 | small nucleolar RNA, H/ACA box 62 | protein synthesis |
| H2ZGU9 | uncharacterized protein (structural constituent of cytoskeleton) | cytoskeleton |
| A0A1W2W7P6 | 26S protease regulatory subunit 6A-B | proteolysis-related |
| E3PQX4 | 40S ribosomal protein S3a | protein synthesis |
| E3PQX0 | ribosomal protein S10 | protein synthesis |
| A0A1W3JML9 | 40S ribosomal protein S15Aa | protein synthesis |
| A0A1W3JP45 | uncharacterized protein (low quality protein) | na |
| E3PQV9 | ribosomal protein L19 | protein synthesis |
| E3PQW8 | ribosomal protein L7 | protein synthesis |
| E3PQV8 | ribosomal protein L18 | protein synthesis |
| E3PQV6 | ribosomal protein L15 **(RP L15)** | protein synthesis |
| A0A1W2WJ63 | coatomer subunit beta | protein synthesis |
| H2Z7H3 | histone H4 | protein synthesis |
| E3PQW1 | ribosomal protein L8 | protein synthesis |
| A0A1W3JUN3 | 40S ribosomal protein S15-like | protein synthesis |
| A0A1W5B8T7 | ATP-dependent RNA helicase DDX3Y isoform X3 | protein synthesis |
| A0A1W2W9L4 | serine/arginine-rich splicing factor 1A **(Ser/Arg rsf 1A)** | protein synthesis |
| E3PQX2 | ribosomal protein S16 | protein synthesis |
| E3PQW2 | ribosomal protein L35a | protein synthesis |
| A0A1W2VZT1 | 60S ribosomal protein L26-like | protein synthesis |
| E3PQX3 | 40S ribosomal protein S18 | protein synthesis |
| A0A1W2W2V1 | 60S ribosomal protein L18a | protein synthesis |
| E3PQV7 | small nucleolar RNA, C/D box 32A | protein synthesis |
| A0A1W2VN77 | 40S ribosomal protein S23 | protein synthesis |
| E3PQW4 | small nucleolar RNA, C/D box 43 | protein synthesis |
| A0A1W2WIZ0 | 60S ribosomal protein L35 **(60S RP L35)** | protein synthesis |
| E3PQW5 | ribosomal protein L4 **(RP L4)** | protein synthesis |
| E3PQV5 | 60S ribosomal protein L13 **(60S RP L13)** | protein synthesis |
| E3PQU9 | small nucleolar RNA C/D box 24 | protein synthesis |
| A0A1W2WNV2 | 60S ribosomal protein L36 | protein synthesis |
| A0A1W2WKT4 | 40S ribosomal protein S9 | protein synthesis |
| E3PQW7 | 60S ribosomal protein L6 | protein synthesis |
| E3PQU7 | ribosomal protein L10-like | protein synthesis |
| Q9BLY0 | intermediate filament protein IF-A | cytoskeleton |
| A0A1W5BDQ6 | ras-related protein Rab-14 | response to stress |
| H2YLK4 | uncharacterized protein (structural constituent of ribosome) | protein synthesis |
| Q9NL97 | G protein alpha subunit | response to stimulus |
| A0A1W2W9L3 | heterogeneous nuclear ribonucleoprotein R isoform X1 | protein synthesis |
| P62153 | calmodulin-A | cytoskeleton |
| Q02508 | protein HGV2 | development-related |
| A0A1W2W7M9 | acyl-coenzyme A oxidase | metabolic-related |
| A0A1W3JUW0 | citrate synthase | energy metabolism |
| A0A1W2W8M0 | long-chain specific acyl-CoA dehydrogenase, mitochondrial-like | energy metabolism |
| A0A1W3JVM8 | creatine kinase, flagellar-like | energy metabolism |
| H2YIN7 | uncharacterized protein (structural constituent of ribosome) | protein synthesis |
| A0A1W2WMD4 | NADH dehydrogenase [ubiquinone] flavoprotein 1, mitochondrial | energy metabolism |
| A0A1W2WHP4 | pyruvate dehydrogenase E1 component subunit alpha | energy metabolism |
| A0A1W3JIR2 | coatomer subunit epsilon | protein synthesis |
| A0A1W3JEH7 | cAMP-dependent protein kinase catalytic subunit alpha isoform X3 | metabolic-related |
| Q9GR89 | putative mitochondrial long-chain 3-ketoacyl-CoA thiolase beta-subunit protein | metabolic-related |
| A0A1W2W6D5 | transitional endoplasmic reticulum ATPase | proteolysis-related |
| A0A1W2VQ55 | axonemal outer arm dynein intermediate chain 1 | cytoskeleton |
| A0A1W3JUC2 | EFHC2 protein isoform X1 | na |
| E3PQV2 | pyruvate dehydrogenase E1 component subunit beta | metabolic-related |
| A0A1W2VN62 | axonemal p66.0 | cytoskeleton |
| A0A1W2W2H3 | growth arrest-specific protein 8 | cytoskeleton |
| A0A1W2WDP7 | cilia- and flagella-associated protein 45-like | cytoskeleton |
| A0A1W2WEI1 | cilia- and flagella-associated protein 58 | cytoskeleton |
| A0A1W2WMX5 | cilia- and flagella-associated protein 20 | cytoskeleton |
| Q8T895 | heat shock protein 40 | response to stress |
| Q8T886 | protofilament ribbon protein | cytoskeleton |
| A0A1W2W944 | dynein light chain | cytoskeleton |
| B5MGP6 | creatine kinase Mt-CK3 | energy metabolism |
| A0A1W2WPT2 | dynein heavy chain 1, axonemal | cytoskeleton |
| A0A1W3JER4 | pf16 protein isoform X1 | cytoskeleton |
| A0A1W2WGR6 | 33 kDa inner dynein arm light chain, axonemal | cytoskeleton |
| A0A1W2WHQ5 | dynein intermediate chain 2, ciliary | cytoskeleton |
| A0A1W2WER0 | tetratricopeptide repeat protein 25 | cytoskeleton |
| A0A1W2WFK4 | tubulin beta chain | cytoskeleton |
| A0A1W3JFS4 | glutamate dehydrogenase | energy metabolism |
| A0A1W2VN58 | tektin A1 | cytoskeleton |
| A0A1W2WFV2 | tektin-1-like | cytoskeleton |
| H2ZDH8 | Uncharacterized protein (GTP biosynthetic process) | protein synthesis |
| Q32TF7 | EFHC1 | cytoskeleton |
| A0A1W2VUY2 | radial spoke protein RSP4/6 | cytoskeleton |
| H2ZGV6 | tubulin alpha chain **(Tubulin ac)** | cytoskeleton |
| F6W1I6 | calcium-transporting ATPase | energy metabolism |
| H2ZKA8 | Uncharacterized protein (microtubule motor activity) | cytoskeleton |
| A0A1W3JBJ7 | dynein heavy chain 8, axonemal | cytoskeleton |
| H2XP87 | uncharacterized protein | na |
| Q6BDZ2 | creatine kinase | energy metabolism |
| A0A1W3JF23 | armadillo repeat-containing protein 4 | cytoskeleton |
| A0A1W2WIW3 | coiled-coil domain-containing protein 173 | protein synthesis |
| A0A1W5B442 | creatine kinase S-type, mitochondrial-like **(CK S-type)** | energy metabolism |
| A0A1W2WK78 | phosphate carrier protein, mitochondrial | energy metabolism |
| Q9UAF3 | glycerin-3-phosphate dehydrogenase | metabolic-related |
| Q25129 | ADT/ATP translocase | energy metabolism |
| A0A1W2W341 | ADP/ATP translocase 3-like | energy metabolism |
| H2ZG57 | tubulin alpha chain | cytoskeleton |
| L7Q2P6 | ADP/ATP translocase 3 | energy metabolism |
| A0A1W2W7Q2 | 2-oxoglutarate dehydrogenase-like, mitochondrial **(2-OD, ml)** | energy metabolism |
| A0A1W5BBF1 | cyclic nucleotide-gated channel rod photoreceptor subunit alpha-like | metabolic-related |
| A0A1W2W5C8 | NADH-ubiquinone oxidoreductase 75 kDa subunit, mitochondrial-like | metabolic-related |
| A0A1W3JEZ2 | succinate dehydrogenase [ubiquinone] flavoprotein subunit, mitochondrial | energy metabolism |
| H2YJV9 | ATP synthase subunit alpha | energy metabolism |
| A0A1W3JCP6 | aconitate hydratase, mitochondrial | energy metabolism |
| H2ZQY8 | ATP synthase subunit beta | energy metabolism |
| H2ZI79 | uncharacterized protein (microtubule motor activity) | cytoskeleton |
| H2Z2A1 | uncharacterized protein (tricarboxylic acid cycle) | energy metabolism |
| A0A024GWF4 | cytochrome c oxidase subunit 1 | energy metabolism |
| H2Z8G3 | uncharacterized protein (ATPase activity, coupled to transmembrane movement of substances) | energy metabolism |
