# Supplementary material for: Proteomic profiling of ascidians as a tool for biomonitoring marine environments
Source: PLoS One. 2019 Apr 9;14(4):e0215005. doi: 10.1371/journal.pone.0215005 (PMC6456167; doi:10.1371/journal.pone.0215005)
Supplement: S2 Table — Abbreviations of the proteins which appear in the column chart (Fig 3B) are specified in parentheses, bold font, following their full name. (DOCX) [file pone.0215005.s002.docx]

| **Protein ID (UniProt)** | **Protein name** | **Biological function** |
| --- | --- | --- |
| H2Y426 | actin, calcium ion binding **(actin cib)** | cytoskeleton |
| O01354 | troponin I | cytoskeleton |
| H2Y9Q3 | catalase | response to stress |
| A0A1W2WQX9 | 14-3-3 protein epsilon-like isoform X1 **(14-3-3 Pe X1)** | response to stress |
| A0A1W2WEM9 | 60S ribosomal protein L23a-like **(60S RP L23a)** | protein synthesis |
| A0A1W2WBP8 | Adenosylhomocysteinase **(AHCY)** | protein synthesis |
| E3PQX8 | seryl-tRNA synthetase **(Ser-tRNA syn)** | protein synthesis |
| F6YMZ5 | ribosome protein | protein synthesis |
| A0A1L3ITD6 | heat shock cognate 71 kDa protein **(HSC71)** | response to stress |
| S0DF50 | cytochrome c oxidase subunit 2 **(Cyt c Ox 2)** | response to stress |
| A0A1W7GYX0 | proton ATPase A **(P-ATPase A)** | metabolic related |
| H2YS57 | enhancer of rudimentary homolog **(ERH)** | protein synthesis |
| A0A1W3JU78 | endoplasmin-like **(Endop-like)** | response to stress |
