# Supplementary figures and images for: Proteomic profiling of ascidians as a tool for biomonitoring marine environments
Source: PLoS One. 2019 Apr 9;14(4):e0215005. doi: 10.1371/journal.pone.0215005 (PMC6456167; doi:10.1371/journal.pone.0215005)

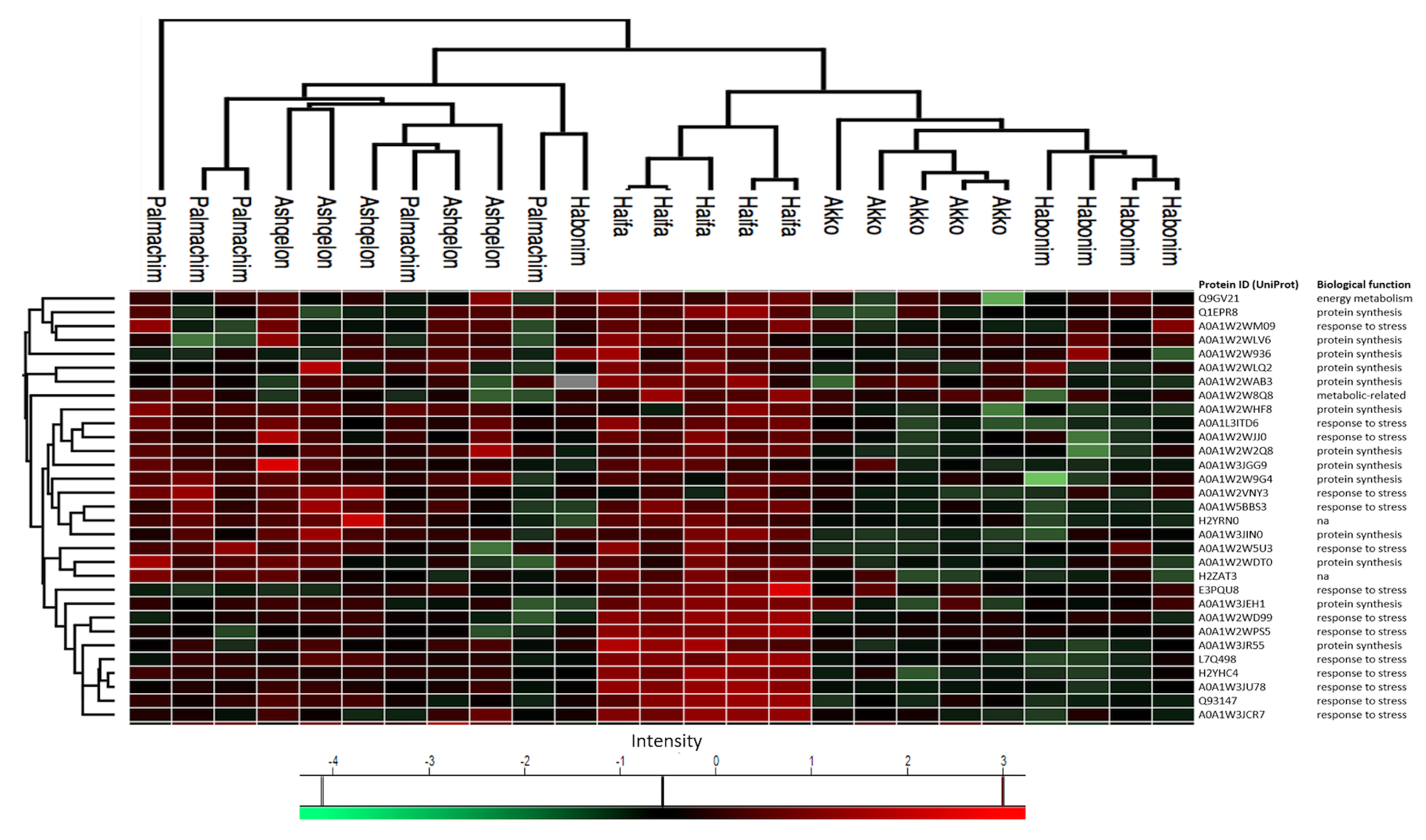

Supplement: S1 Fig — Protein names are listed in S1 Table. na = not available, assigned to proteins for which data on their biological functions could not be obtained. (TIF) [file pone.0215005.s003.tif]
